# High-Performance Fiber-Shaped Supercapacitors Enabled by Polyaniline @ MXene Ti_3_C_2_T_x_ Hybrid Graphene Aerogel Fiber

**DOI:** 10.3390/gels12080690

**Published:** 2026-08-03

**Authors:** Ran Jin, Qingquan Xue, Yang Zhang

**Affiliations:** 1 College of Artificial Intelligence, Ningbo University of Finance and Economics, Ningbo 315175, China; ran.jin@163.com; 2 Zhejiang Collaborative Innovation Center for Full-Process Monitoring and Green Governance of Emerging Contaminants, Interdisciplinary Research Academy (IRA), Zhejiang Shuren University, Hangzhou 310015, China; 3 National Engineering Lab for Textile Fiber Materials & Processing Technology, Zhejiang Sci-Tech University, Hangzhou 310018, China

**Keywords:** graphene aerogel fiber, covalent bond, Ti_3_C_2_T_x_ MXene, polyaniline, fiber-shaped supercapacitors

## Abstract

Developing fiber-shaped supercapacitors (FSCs) with high capacitance, high energy density, and exceptional rate performance is crucial for reliable, stable and high-performance wearable electronics. Here, a polyaniline (PANI) @ MXene Ti_3_C_2_T_x_/graphene (PTG) aerogel fiber was rationally fabricated via a confined hydrothermal method followed by freeze-drying treatment. The graphene sheets construct the skeleton of the aerogel fiber, which possesses a porous and interlinked structure, providing interconnected diffusion channels and a high specific surface area that promote electrolyte migration and abundant ion adsorption sites. Moreover, the covalent modification between PANI nanoparticles and Ti_3_C_2_T_x_ sheets can significantly improve interfacial coupling and provide abundant redox sites, resulting in a reduced energy barrier of electron transfer, good interfacial stability and superior H^+^ storage capability. As a consequence, the PTG aerogel fiber electrode delivers an excellent specific mass capacitance of 484.8 F g^−1^, impressive rate properties (244.4 F g^−1^ at 10 A g^−1^) and exceptional cycle ability (85.2% after 5000 cycles). Additionally, the fabricated symmetrical FSC exhibits considerable electrochemical performance, including high capacitance and good energy density. This work depicts a novel route to prepare a graphene fiber-based electrode for high-performance FSCs in an intelligent wearable system.

## 1. Introduction

Wearable energy storage electronics featuring high efficiency, light weight, and miniaturization are in high demand for next-generation wearable electronics, including smart healthcare systems, Internet of Things hardware, and artificial electronic skin [1,2,3,4]. Compared with two-dimensional (2D) devices, one-dimensional energy devices exhibit tremendous advantages in textile integration, air permeability, and mechanical deformability. Among these devices, fiber-shaped supercapacitors (FSCs) are regarded as promising power sources, owing to their safe working conditions, fast charge/discharge performance, and excellent long cycling lifespans [5,6,7]. Yet, the fiber electrode for the prevailing FSC—with circuitous diffusion networks, unstable interface interaction, and discontinuous electron transfer pathways—inevitably results in poor electrochemical performance. Thus, it is urgent to fabricate fiber electrodes with optimized porous architectures, high conductivity and strong interfacial coupling for high-performance FSCs.

Graphene, a family of 2D carbon-based materials, has attracted widespread interest in the FSC field due to its remarkable conductivity, large specific surface area, and superior electrochemical properties [8,9]. The flexible graphene fibers, assembled via the wet spinning process [10], hydrothermal method [11], electrochemical deposition method [12], and other approaches, have been utilized in FSCs and have delivered impressive electrochemical and mechanical properties. For instance, a locally aligned graphene fiber was developed by a non-liquid-crystal spinning method, achieving a specific mass capacitance of 279 F g^−1^ [13]. However, because of the layer interaction between the adjacent graphene sheets, the graphene fiber is inclined to form a compact structure, resulting in slow ion diffusion kinetics and insufficient active sites. Constructing porous structures has been verified as an effective approach to prevent graphene nanosheets from restacking and supply abundant active sites. Among various structural designs, the aerogel structure has received much attention on account of a large accessible surface area, interconnected architectures and unrestricted diffusion channels [14,15]. Graphene can easily form a three-dimensional (3D) network by self-assembly of 2D graphene oxide sheets under the hydrothermal condition and the freeze-drying treatment, which guarantees ion accessibility and reaction rapidity [16]. Even so, the graphene fiber-based FSC is mainly restricted by limited redox active sites and low energy density. Thus, rationally designing porous graphene fibers that combine the strengths of graphene fibers and pseudo-capacitive materials is an effective approach to obtain superior electrochemical performance.

MXene (M_n+1_X_n_T_x_), a burgeoning 2D transition metal carbide, serves as a promising candidate for electrochemical energy storage because of its overwhelming electrical conductivity, unrivaled energy storage capability, plentiful surface groups and good mechanical performance [7]. Introducing MXene into graphene fibers can form a layer-by-layer stacked structure, which accelerates electron transfer and boosts both energy and power performance. For instance, a large MXene flake was adopted to improve ion accessibility to the internal pores of fibers, realizing excellent energy density and high power density [17]. Additionally, well-aligned MXene/graphene hybrid fibers with high electrical conductivity and superior flexibility were assembled for fiber-shaped energy storage devices [18]. In addition, introducing conductive polymers and functional materials, such as polyaniline (PANI) [19], metal-organic frameworks (MOFs) [20], metal hydroxide [21], and so on, is a promising method to enhance charge storage capability and optimize ion migration kinetics. For example, PANI arrays grown on partially reduced graphene oxide to form hybrid fibers with highly stable skeletons were prepared through an in situ polymerization method, delivering a high capacitance of 50.2 F cm^−3^ at a huge current density (60 mA cm^−3^) [22]. However, the above-mentioned graphene-based electrodes are still confronted with an ineluctable challenge. The infirm and unstable interfacial interactions between active materials and the conductive substrate result in sluggish charge transmission, inferior ion accessibility and limited capacitance.

In this work, we fabricated a porous hierarchical PANI @ MXene Ti_3_C_2_T_x_ hybrid graphene (PTG) aerogel fiber via a confined hydrothermal method followed by freeze-drying treatment. The graphene aerogel fiber with a porous conductive network provides enough pores for fast ion diffusion and a large surface area for sufficient ion adsorption. More importantly, the covalent bonds between PANI and Ti_3_C_2_T_x_ generated during the in situ polymerization reaction can not only build a stable heterogeneous interface to boost electron migration and strengthen structural stability during charge/discharge processes, but also create a synergistic effect that provides abundant redox active sites and improves energy storage capacity. As a result, the synthesized PTG aerogel fiber is equipped with outstanding specific capacitance (484.8 F g^−1^ at 1 A g^−1^), impressive cycling performance (85.2% capacitance retention for 5000 cycles) and favorable rate property (244.4 F g^−1^ at 10 A g^−1^). Additionally, the assembled symmetrical FSC achieves a high capacitance of 275.6 F g^−1^ at 1 A g^−1^ and exceptional energy density of 3.44 Wh kg^−1^. More importantly, the FSC can be connected to electronic devices or even integrated into textiles and work stably, which endows it with potential for future utilization in flexible portable smart systems.

## 2. Results and Discussion

**Figure 1:**
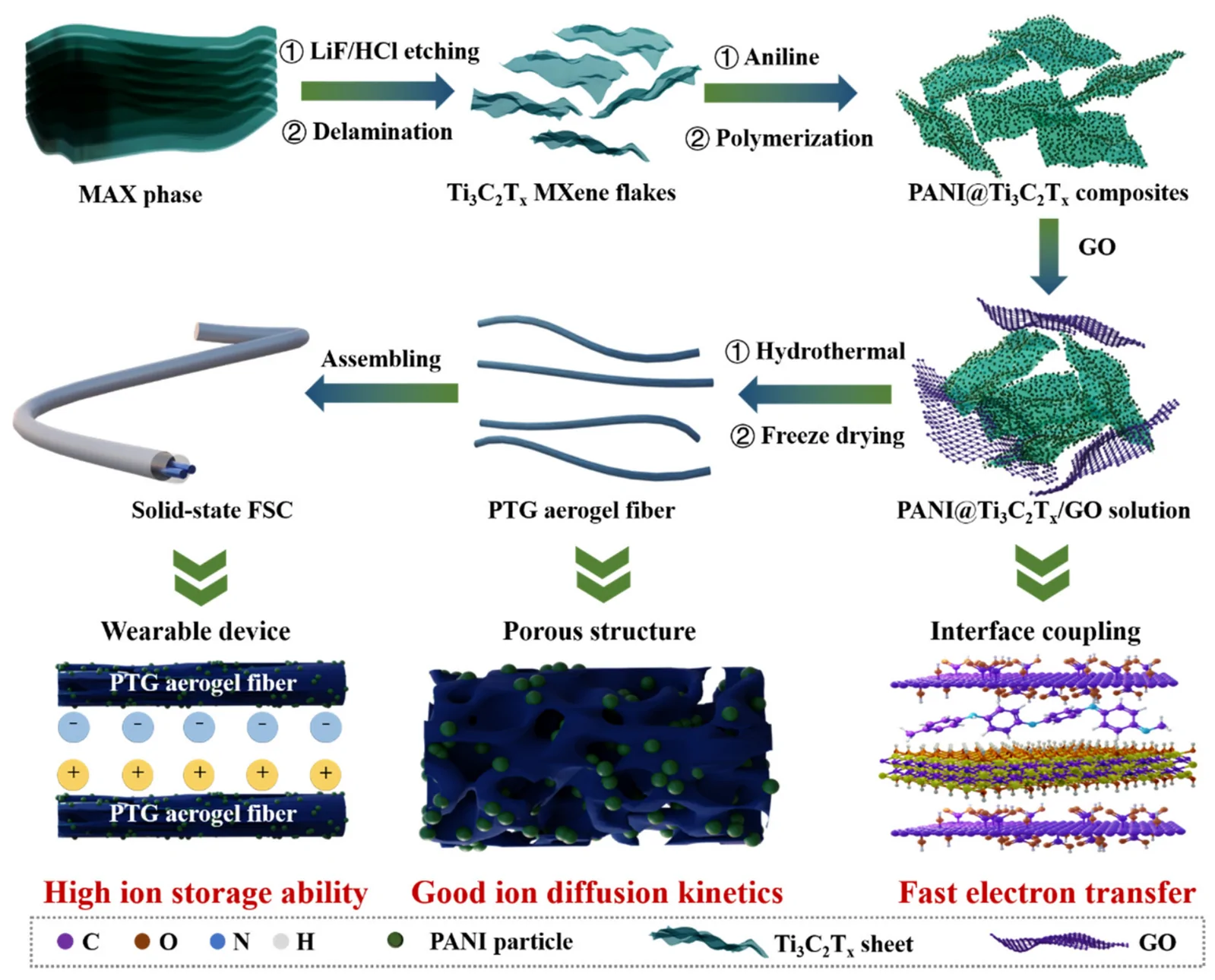
Schematic illustration for the stepwise preparation of a PTG aerogel fiber and solid-state FSC.

Figure 1 shows the schematic for fabricating PTG aerogel fibers and assembling FSC devices. Firstly, Ti_3_C_2_T_x_ flakes were obtained using the LiF/HCl etching method combined with delamination treatment. Subsequently, the PANI nanoparticles were anchored on Ti_3_C_2_T_x_ flakes by the in situ fabrication process to produce PANI@Ti_3_C_2_T_x_ composites. Then, as-prepared PANI@Ti_3_C_2_T_x_ composites were mixed with the GO solution at room temperature. Due to the physical interaction (hydrogen bonds between PANI, Ti_3_C_2_T_x_ and GO) and the chemical interaction (covalent bonds between PANI and Ti_3_C_2_T_x_), the PANI@Ti_3_C_2_T_x_/GO solution formed favorable interfacial coupling, which could construct a continuous conductive network and accelerate electron transfer [22]. After that, the PANI@Ti_3_C_2_T_x_/GO solution was injected into a polypropylene tube for a hydrothermal reaction before undergoing a freeze-drying treatment. The obtained PTG aerogel fiber presents 3D frameworks and porous networks which are beneficial for ion diffusion and H^+^ adsorption. Finally, the solid-state symmetrical FSC can be assembled for wearable systems.

**Figure 2:**
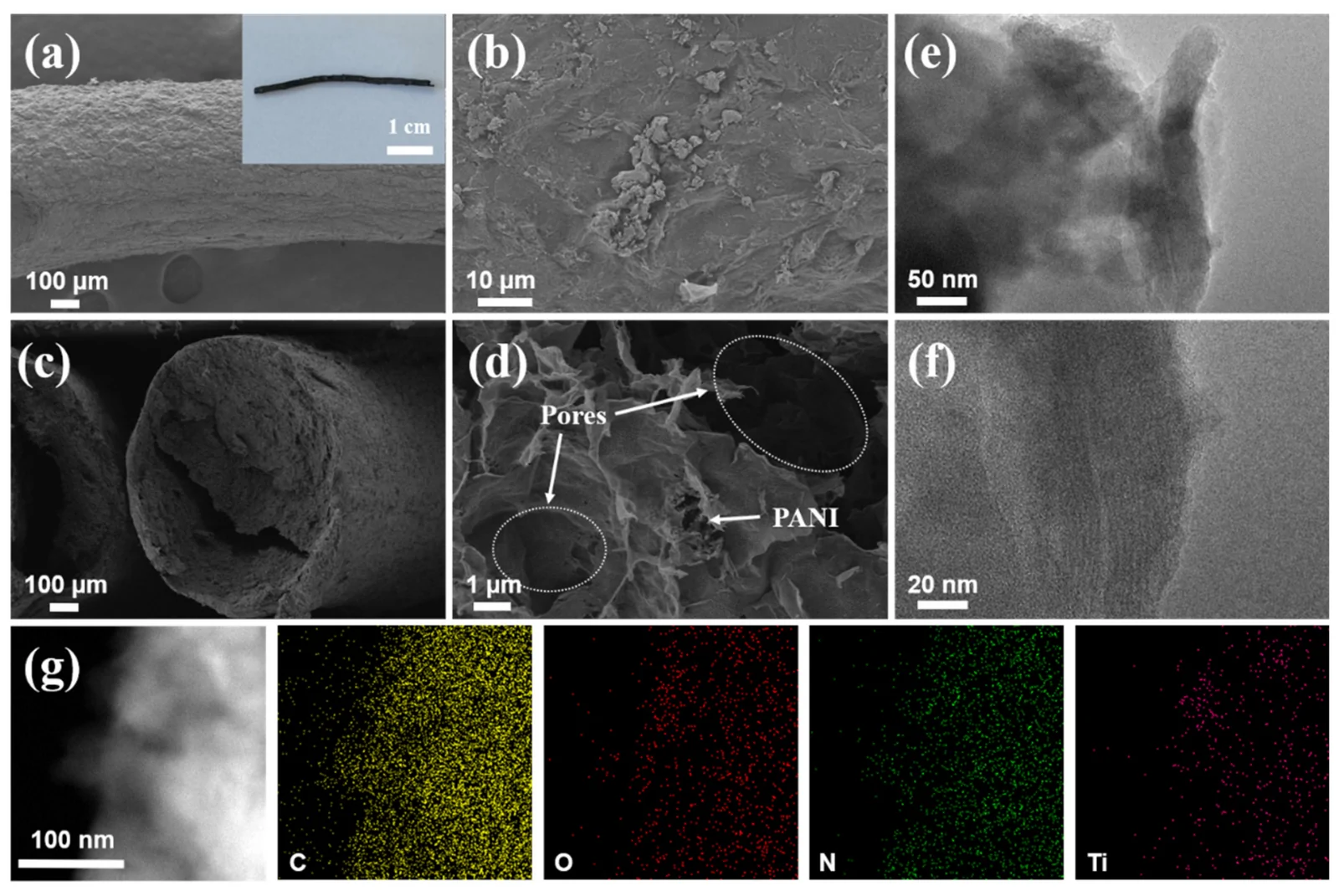
(**a**,**b**) Surface and (**c**,**d**) cross-sectional SEM images of a PTG aerogel fiber. (**e,f**) TEM images of a PTG aerogel fiber. (**g**) C, O, N, Ti elements’ distribution of a PTG aerogel fiber.

SEM and TEM characterizations were carried out to characterize the morphological architectures of electrodes. In Figure S1, a smooth structure for the rGO fiber can be observed via surface SEM images. After adding Ti_3_C_2_T_x_ and PANI, rough structures with wrinkles and defects can be observed on the Ti_3_C_2_T_x_/rGO (TG) fiber, PTG fiber and PTG aerogel fiber (Figure 2a,b and Figures S2 and S3). Furthermore, hierarchical porous structures are verified by the high-resolution SEM observations in Figure 2c,d. Obviously, thanks to the freeze-drying treatment, abundant voids distributed inside the PTG aerogel fiber create interconnected channels and supply sufficient active sites for ion migration and storage, respectively. In addition, pore walls composed of stacked rGO and Ti_3_C_2_T_x_ nanosheets form due to physical interactions between these two-dimensional layers, offering fast pathways for electron transfer. In contrast, the cross-sectional SEM images for rGO fiber, TG fiber and PTG fiber show compact dense frameworks, impeding ion diffusion inside the fibers and blocking active sites on the nanosheets (Figures S4–S6). Moreover, the TEM images in Figure 2e and 2f display lattice spacings of 0.33 nm and 1.41 nm, belonging to the (002) planes of rGO and Ti_3_C_2_T_x_, respectively [17]. The mapping images of N, O, C and Ti collected from selected regions clearly demonstrate the uniform distribution of these elements (Figure 2g).

**Figure 3:**
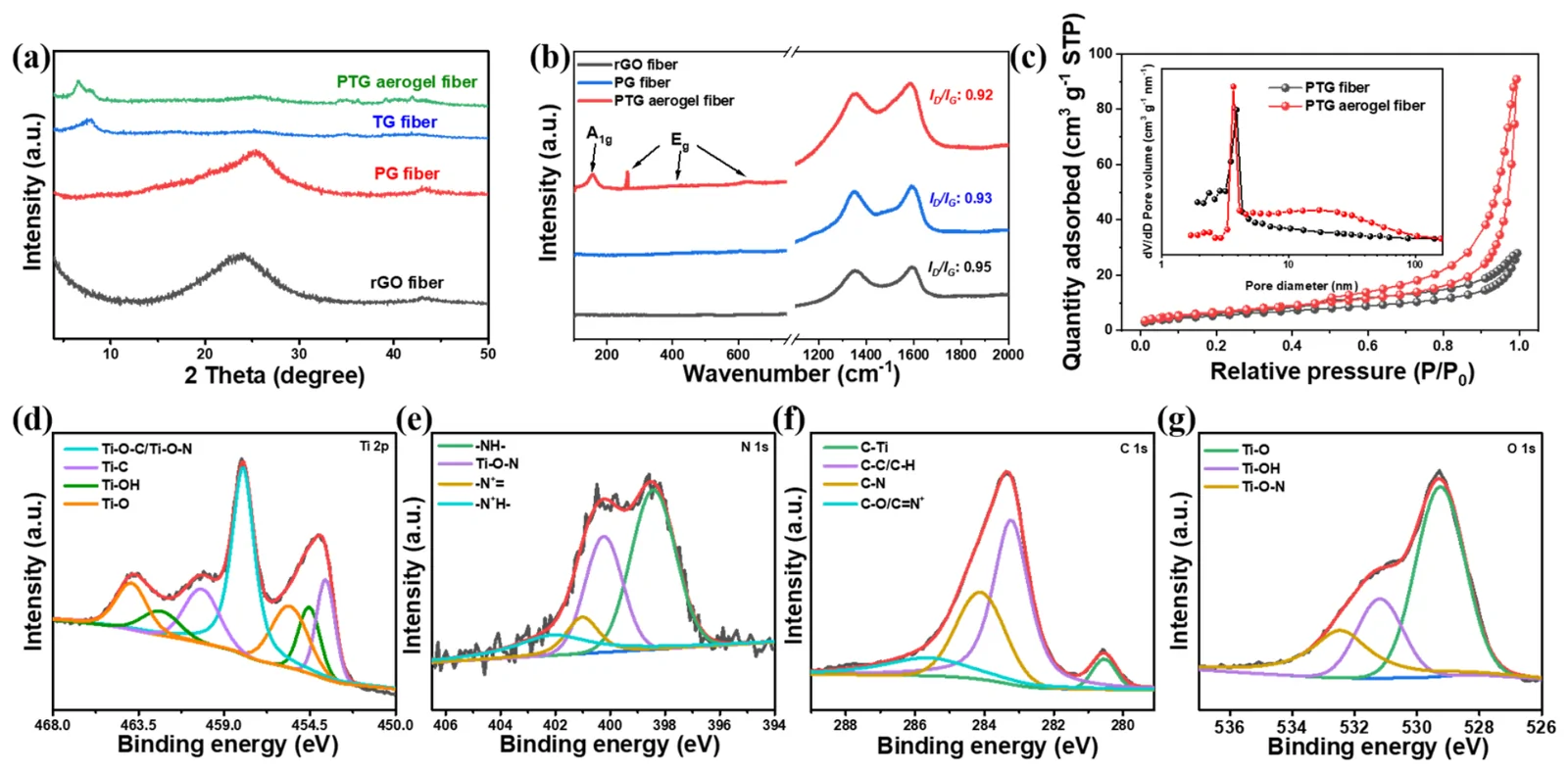
(**a**) XRD patterns of the rGO fiber, TG fiber, PTG aerogel fiber. (**b**) Raman spectra of the rGO fiber, PG fiber and PTG aerogel fiber. (**c**) Nitrogen adsorption–desorption isotherms and pore diameter distribution of the PTG fiber and PTG aerogel fiber. The XPS high-resolution spectra of (**d**) Ti 2p, (**e**) N 1s, (**f**) C 1s and (**g**) O 1s.

Figure 3a portrays the XRD patterns of rGO fiber, TG fiber, PANI/rGO (PG) fiber and PTG aerogel fiber. A broad peak at around 24.6° assigned to the (002) plane of rGO is detected in the spectra of rGO and PG fibers [23]. Upon incorporation of Ti_3_C_2_T_x_, a characteristic peak at around 7.6°, originating from the (002) plane of Ti_3_C_2_T_x_, becomes dominant for the TG fiber. Furthermore, the (002) plane of the PTG aerogel fiber shifts to 6.4°. This shift verifies that PANI intercalates into the interlayers of Ti_3_C_2_T_x_ flakes during in situ polymerization, enlarging interlayer spacing and accelerating ion diffusion [24].

Figure 3b presents the Raman spectra of rGO fiber, PG fiber and PTG aerogel fiber. The pure rGO fiber exhibits two representative peaks at 1351 cm^−1^ and 1590 cm^−1^ [5]. After adding the PANI and Ti_3_C_2_T_x_ flakes, the I_D_/I_G_ slightly declines from 0.95 to 0.92 and 0.93, which can be ascribed to interfacial interactions among rGO, Ti_3_C_2_T_x_ and PANI. Moreover, distinct Ti_3_C_2_T_x_ characteristic peaks emerge in the PTG aerogel fiber spectrum. The A_1g_ peak at 163 cm^−1^ corresponds to the out-of-plane vibrations of titanium and carbon atoms, respectively. The E_g_ peaks located at 263, 405, and 625 cm^−1^ originate from the in-plane vibrations of titanium, carbon, and functional groups, respectively. The existence of these characteristic peaks proves that Ti_3_C_2_T_x_ flakes retain their intact structure throughout fiber fabrication [25].

The surface area and pore architectures of the PTG fiber and PTG aerogel fiber were surveyed via N_2_ (77 K) adsorption/desorption isotherms and the pore diameter distribution, as shown in Figure 3c. Both fibers deliver type II isotherms with the obvious H3 hysteresis loop, demonstrating the features of mesopores and micropores [26]. Moreover, the pore size distribution indicates that the PTG aerogel fiber shows increased porosity in the mesopore range compared with the PTG fiber, which can be ascribed to the porous structure design. As a result, its specific surface area and pore volume rise from 19.86 to 25.23 m^2^ g^−1^ and 0.034 to 0.087 cm^3^ g^−1^, respectively, which can be attributed to the consequence of the customized 3D networks and heterogeneous structures, leading to a good aggregation-resistant property, exceptional ionic transport kinetics and favorable electron transfer capabilities.

XPS confirmed the formation of chemical bonds in the PTG aerogel fiber. The results show that Ti, C, N and O elements exist in the PTG aerogel fiber (Figure S7). The Ti 2p spectrum of typical peaks at binding energies of 460.28/453.68, 462.48/454.58, 463.88/455.58 and 457.98 eV is attributed to the Ti–C, Ti–OH, Ti–O and Ti–O–N bonds, respectively (Figure 3d) [27]. The chemical bonds between Ti_3_C_2_T_x_ and PANI can construct a strong interfacial interaction, leading to fast electron transfer and cycling stability [28]. Four peaks centered at 280.58, 283.28, 284.18, and 285.68 eV can be identified in the C 1s spectrum, attributed to Ti-C, C-C, C-N, and C-O/C=N, respectively (Figure 3e) [29]. Four characteristic peaks at 398.38, 400.18, 400.98 and 401.98 eV in the high-resolution N 1s spectrum are classified as -NH-, Ti-O-N, -N^+^= and -N^+^H-, respectively (Figure 3f) [30]. As shown in Figure 3g, three peaks in the O 1s spectrum generated by fitting at 532.48, 531.18, and 529.28 eV are attributed to Ti-O-N, Ti-OH and Ti-O bond, respectively [31].

**Figure 4:**
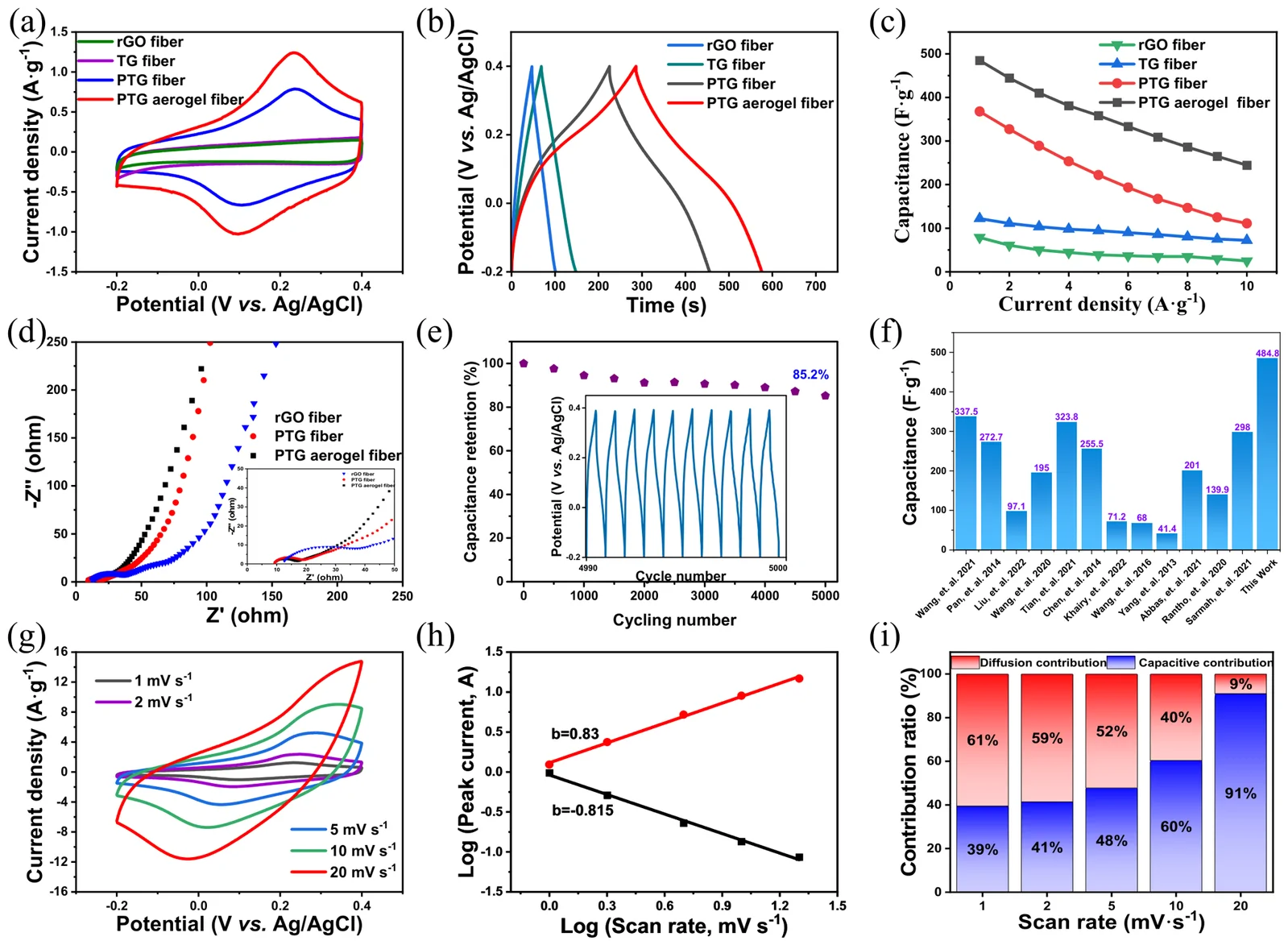
(**a**) CV curves and (**b**) GCD profiles of rGO fiber, TG fiber, PTG fiber and PTG aerogel fiber. (**c**) GCD profiles of a PTG aerogel fiber at 1–10 A g^−1^. (**d**) Rate performance of rGO fiber, TG fiber, PTG fiber and PTG aerogel fiber. (**e**) Long-term cycling property of a PTG aerogel fiber at 10 A g^−1^. (**f**) Comparison of mass capacitance of a PTG aerogel fiber with previously reported electrodes [34,35,36,37,38,39,40,41,42,43,44,45]. (**g**) CV curves at a scan rate ranging from 1 to 20 mV s^−1^, (**h**) the relationship of peak current density with sweep rate, and (**i**) capacitive contributions of a PTG aerogel fiber at a scan rate of 1–20 mV s^−1^.

The electrochemical properties of fiber electrodes were evaluated with the three-electrode system. Figure 4a shows CV curves of the rGO fiber, TG fiber, PTG fiber and PTG aerogel fiber at 1 mV s^−1^. Distinct redox peaks appear in the PTG fiber and PTG aerogel fiber, indicative of typical pseudo-capacitance originating from reversible redox transitions of PANI between its reduced and fully oxidized states [32]. Additionally, PTG aerogel fiber is equipped with the largest closed area of CV curves, compared with rGO fiber, TG fiber and PTG fiber at the same scan rate, suggesting the higher ion storage capability. Figure 4b shows the GCD curves of rGO fiber, TG fiber, PTG fiber and PTG aerogel fiber at the current density of 1 A g^−1^. Different from the rGO fiber, TG fiber and PTG fiber, PTG aerogel fiber possesses a longer discharge time, indicating its good energy storage ability and exceptional electrochemical properties. Moreover, an apparent curvature can be found on the PTG fiber and the PTG aerogel fiber, illustrating the pseudo-capacitive behavior from the redox reaction of PANI nanoparticles.

Figure S8 displays the GCD profiles of a PTG aerogel fiber at the current density ranging from 1 to 10 A g^−1^. Nearly triangular GCD profiles of hybrid fiber electrodes at various current densities demonstrate favorable reversibility and high coulombic efficiency. Figure 4c exhibits the rate performance of each electrode. The PTG aerogel fiber possesses the maximum capacitance of 484.8 F g^−1^, while PTG and TG fibers show capacitances of 372.9 and 80.8 F g^−1^, respectively (Figures S9–S11). In addition, the capacitance of fiber electrodes decreases with the improvement in current density, attributed to the limited ion diffusion kinetics and insufficient charge transport rates. Furthermore, PTG aerogel fiber portrays a higher capacitance of 244.4 F g^−1^ than those of rGO, TG and PTG fibers at a current density of 10 A g^−1^, which originates from the porous structure, interface coupling and pseudo-capacitance enhancement providing abundant redox active sites, interconnected diffusion channels and successive electron transfer pathways.

Figure 4d shows the EIS curves of the rGO fiber, the PTG fiber and the PTG aerogel fiber. Obviously, the PTG aerogel fiber exhibits lower charge-transfer resistance and Warburg impedance than those of rGO fiber and PTG fiber, suggesting that the porous structure and interconnected networks can significantly facilitate ion diffusion and electron transfer [33]. Furthermore, the long-term cycling property of the PTG aerogel fiber is detected at 10 A g^−1^. The PTG aerogel fiber electrode still maintains a high capacitance retention of 85.2% after 5000 cycles (Figure 4e). The exceptional cycling performance can be attributed to the covalent interaction between the PANI chains and Ti_3_C_2_T_x_ flakes and the synergistic effect between the heterogeneous interface via hydrogen bonds. Accordingly, the designed PTG aerogel fiber exhibits superior electrochemical performance, surpassing many reported flexible electrodes, including PANI/MXene (V_2_C) (337.5 F g^−1^) [34], carbon nanotubes/PANI composite (272.7 F g^−1^) [35], MXene@ZIF-67 (97.10 F g^−1^) [36], rGO hybrid MXene fiber (195 F g^−1^) [37], PANI@CNT fiber (323.8 F g^−1^) [38], elastic fiber/CNTs/PANI (255.5 F g^−1^) [39], PANI@GO-coated silk fabric (71.2 F g^−1^) [40], N-doped rGO/PAA/PANI composites (68 F g^−1^) [41], CNT sheets coated on elastic fiber (41.4 F g^−1^) [42], ZnO@CFT (201 F g^−1^) [43], C-Fe/PANI/Ni-GF (139.9 F g^−1^) [44] and MoS_2_/rGO/PEDOT/NF (298 F g^−1^) [45] (Figure 4f).

To explore the H^+^ diffusion process of the PTG aerogel fiber electrode, the voltammetric sweep rate dependence is investigated (Figure 4g). The equation i = a v^b^, was applied, where i is peak current, v is scan rate and a, b are adjustable parameters [46]. As illustrated in Figure 4h, the b values of peaks can be calculated as 0.83 and 0.815, respectively, which indicates that energy storage behavior in the PTG aerogel fiber is mainly dominated by the capacitive behavior. In addition, the equation: i = k_1_v + k_2_v^1/2^, is used to calculate the capacitive contribution ratio of a PTG aerogel fiber, where k_1_v and k_2_v^1/2^ are capacitive-controlled contribution and diffusion-controlled contribution, respectively [47]. At the scan rate of 1 mV s^−1^, the capacitive contribution ratio of the PTG aerogel fiber is 39%. After increasing the scan rate to 20 mV s^−1^, this value increases to 91%, demonstrating that capacitive behavior plays a dominant role in the energy storage process for the PTG aerogel fiber electrode (Figure 4i and Figures S12–S16).

**Figure 5:**
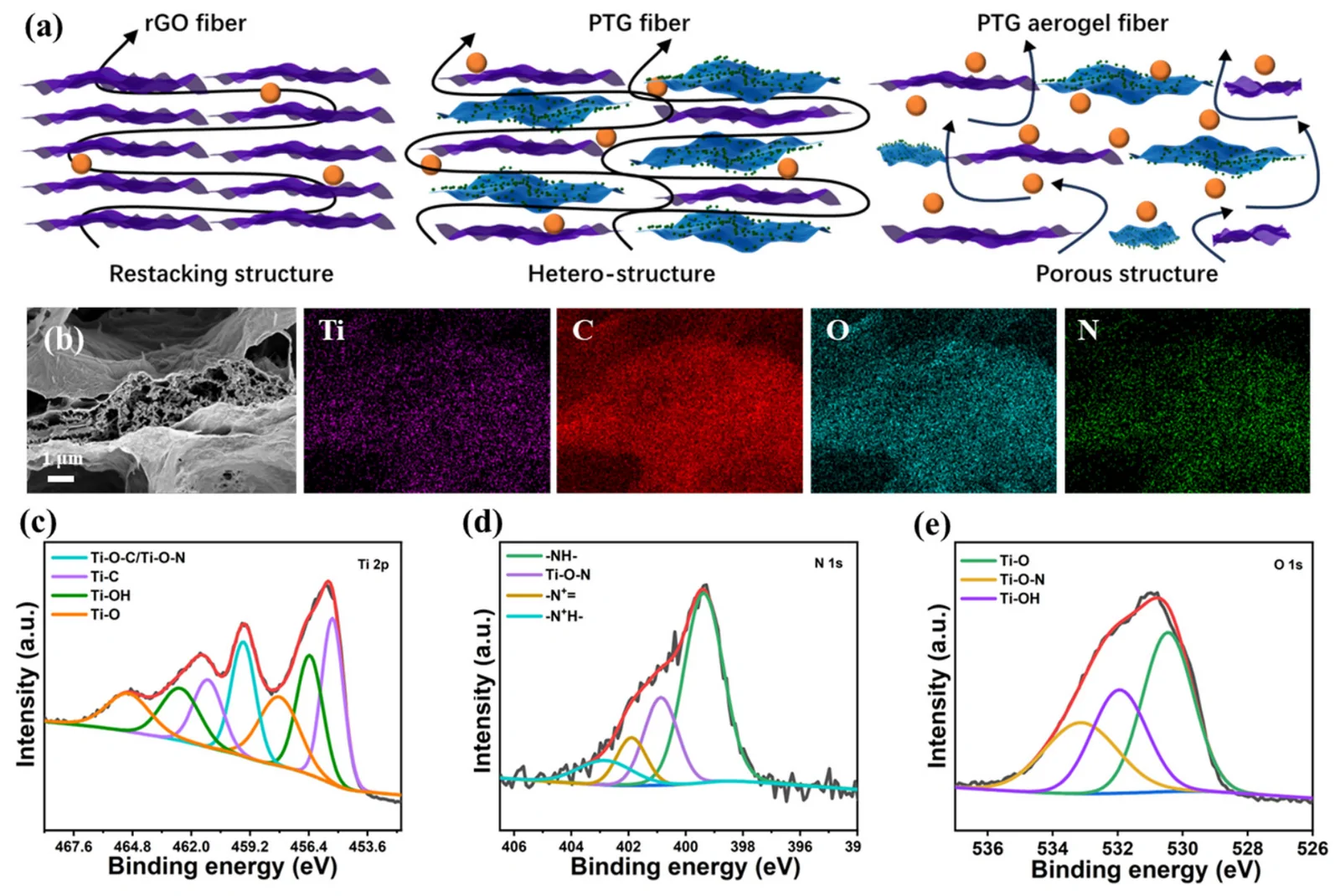
(**a**) The energy storage mechanism of rGO fiber, PTG fiber and PTG aerogel fiber. (**b**) The cross-sectional SEM image and element distribution of a PTG aerogel fiber after cycling. High-resolution XPS spectra of (**c**) Ti 2p, (**d**) N 1s and (**e**) O 1s.

Given the porous structure, interfacial coupling and rich redox active sites, the PTG aerogel fiber possesses outstanding electrochemical properties (Figure 5a). Owing to the π-π interaction and spontaneous layer-by-layer restacking effect, the rGO fiber presents a tight structure, causing sluggish ion diffusion and limited H^+^ adsorption sites. After adding PANI and Ti_3_C_2_T_x_, the PTG fiber portrays hetero-structures and abundant redox active sites, exhibiting good capability in energy storage compared with pure rGO fiber. When a 3D porous structure is constructed for the PTG aerogel fiber, the interconnected porous channels and huge specific surface areas provide a pathway for good ion diffusion kinetics. Moreover, the covalent linkage between PANI and Ti_3_C_2_T_x_ endows with stable interfacial interactions, leading to rapid electron migration and excellent long-term cycling performance.

After that, the cross-sectional SEM image of the PTG aerogel fiber is performed to investigate the structure variation after cycling (Figure 5b). Obviously, the PTG aerogel fiber still maintains a porous structure and its architecture without collapse after the repeated charge/discharge process. In addition, the distribution of elements in the fiber confirms that the PANI, MXene and rGO flakes are homogeneously distributed in the aerogel fiber. The result illustrates that the PTG aerogel fiber exhibits stable skeleton frameworks and good interfacial coupling. Additionally, the XPS spectra are applied to test the chemical bond state of the PTG aerogel fiber after cycling (Figure 5c–e). Notably, the separated bonds of Ti 2p, N 1s and O 1s spectra present without significant change after cycling, illustrating that the interface interaction and molecule structure of Ti_3_C_2_T_x_, PANI and rGO present good stability.

**Figure 6:**
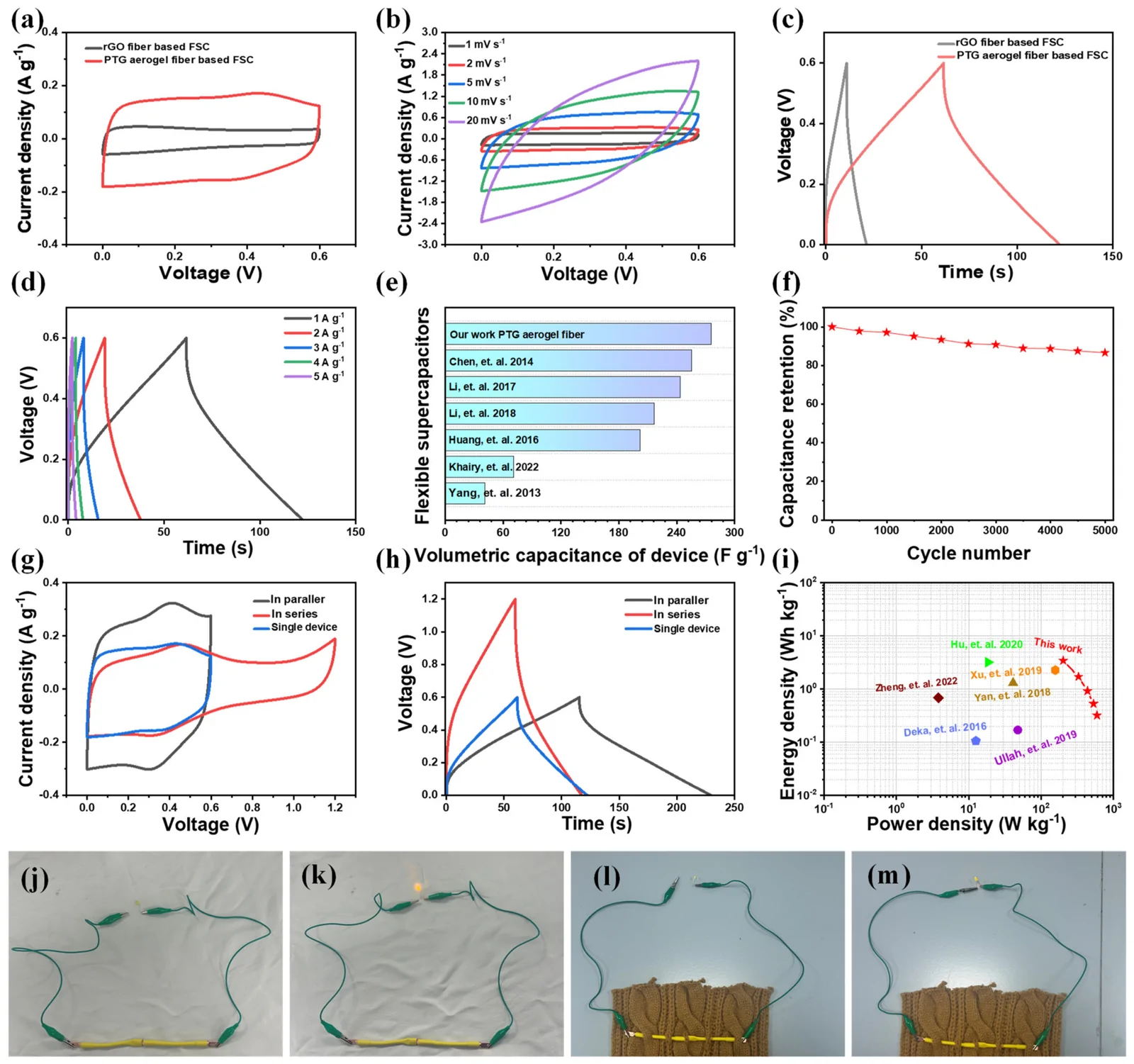
(**a**) CV curves of rGO fiber and PTG aerogel fiber-based FSC. (**b**) CV curves of the PTG aerogel fiber-based FSC at 1–20 mV s^−1^. (**c**) GCD curves of rGO fiber and PTG aerogel fiber-based FSC. (**d**) GCD profiles at 1–5 A g^−1^ of the PTG aerogel fiber-based FSC. (**e**) Comparison of capacitance with previously reported flexible devices [39,40,42,48,49,50]. (**f**) Long-term cycling of the PTG aerogel fiber-based FSC. (**g**) CV curves and (**h**) GCD curves for a single as-prepared PTG aerogel fiber-based FSC and two connected devices in series and parallel. (**i**) Ragone plot of the PTG aerogel fiber-based FSC [51,52,53,54,55,56]. Photograph of the PTG aerogel fiber-based FSC (**j**,**k**) driving an LED and (**l**,**m**) integrating a fabric as a power source.

A quasi-solid-state symmetrical FSC with a parallel structure using PTG aerogel fiber as electrode and 1 M H_2_SO_4_/polyvinyl alcohol (PVA) gel as gel electrolyte is assembled to evaluate its application potential on a wearable system. As depicted in Figure 6a, the CV curves of FSC based on the electrode of rGO fiber and PTG aerogel fiber are displayed. Significantly, PTG aerogel fiber-based FSC possesses a larger closed area, which illustrates higher energy storage capability. Furthermore, as demonstrated in Figure 6b and Figure S17, both fiber-based devices present good electrochemical reversibility because of the well-maintained CV curves at high scan rates. Additionally, the GCD profiles of rGO fiber and PTG aerogel fiber-based FSCs with a nearly symmetrical triangular profiles suggest that they possess excellent cycle properties (Figure 6c). The specific mass capacitance of the PTG aerogel fiber-based FSC is 275.6 F g^−1^ at 1 A g^−1^, surpassing the capacitance of rGO fiber-based FSC (40 F g^−1^). At the current density of 5 A g^−1^, PTG aerogel fiber-based FSC still shows high capacitance of 25.6 F g^−1^ (Figure 6d). Nevertheless, the rGO fiber-based FSC decreases to 1.7 F g^−1^ at 3 A g^−1^ (Figure S18). The impressive electrochemical performance of the PTG aerogel fiber-based FSC can be attributed to the stable interfacial interaction, sufficient ion diffusion channels and successive conductive frameworks. Satisfactorily, the high capacitance of the PTG aerogel fiber-based FSC is superior to previously flexible devices, such as CNTs/PANI/elastic fiber (255.5 F g^−1^) [39], hydrothermally activated graphene fiber (244 F g^−1^) [48], MCNF- mesoporous carbon nanofiber (216.6 F g^−1^) [49], PPy/MWCNT/cotton fabric (201.99 F g^−1^) [50], PANI@GO-coated silk fabric (71.2 F g^−1^) [40], and CNT-coated elastic fiber (41.4 F g^−1^) [42] (Figure 6e and Table S2). Moreover, the FSC shows remarkable cycling stability, retaining 86.6% capacitance after 5000 cycles at 5 A g^−1^ (Figure 6f).

To satisfy the voltage requirements of practical applications, the FSC is connected in series or parallel (Figure 6g,h). By connecting two FSCs in series, the output voltage is increased from 0.6 V to 1.2 V, achieving a doubling of voltage without sacrificing the charge/discharge time relative to a single device. At the same current density and operating voltage, the two series-connected devices show a charge/discharge time twice as long as that of a single FSC. In addition, Ragone plot analysis illustrates the superior energy storage performance of FSC, delivering an energy density of 3.44 Wh kg^−1^ at a power density of 202.4 W kg^−1^, outperforming previously set out values in the literature, such as carbon fiber/PET (0.106 Wh kg^−1^) [51], PPy-MXene (1.3 Wh kg^−1^) [52], CNT-NiCo_2_O_4_ (3.2 Wh kg^−1^) [53], MCNT/bamboo joint (2.27 Wh kg^−1^) [54], PANI/MXene@polyester (0.69 Wh kg^−1^) [55], and CCY-MnO_2_ (0.17 Wh kg^−1^) [56] (Figure 6i). The above-mentioned results indicate good scalability of the FSCs. In practical applications, as illustrated in Figure 6j,k, a light-emitting diode (LED) can be driven by connected FSCs in series stably. Moreover, we embedded two devices into the fabric successfully, which can also power an LED reliably (Figure 6l,m).

## 3. Conclusions

In summary, we prepared a porous PTG aerogel fiber via the confined hydrothermal method followed by freeze-drying treatment. The graphene conductive frameworks provide interconnected electrolyte diffusion channels and successive electron migration pathways. Moreover, introducing PANI and Ti_3_C_2_T_x_ provides adequate redox sites and an exceptional capacitance of 484.8 F g^−1^. More importantly, the chemical bonds between PANI and Ti_3_C_2_T_x_ render a stable interfacial interaction and good cycling properties. Additionally, the assembled FSC delivers enhanced energy storage capability, high energy density, and excellent integration ability. Considering the requirement of scalability, functionality, and long-term cycling property for FSC, the PTG aerogel fiber can promote the further development of the device in wearable and portable electronic devices.

## 4. Materials and Methods

### 4.1. The Fabrication of a PTG Aerogel Fiber

A PTG aerogel fiber was synthesized by a confined hydrothermal method followed by freeze-drying treatment. The specific process was as follows: (1) The PANI nanoparticles @ Ti_3_C_2_T_x_ sheets (PT) were obtained via an in situ chemical polymerization method. Typically, 0.7 g aniline (Aladdin, AR, 99.5%, Shanghai, China) monomers and 30 mL 1 M hydrochloric acid (HCl) (Shanghai Lingfeng Chemical Reagent Co., Ltd., Shanghai, China) solution were slowly added to the 1 mL Ti_3_C_2_T_x_ solution (100 mg mL^−1^) (11 Technology Co., Ltd., Beijing, China) under stirring for 30 min in an ice bath. After that, 0.42 g ammonium persulfate ((NH_4_)_2_S_2_O_8_) (Aladdin, AR, 98%, Shanghai, China) was quickly poured into the above-mentioned solution, stirred in an ice bath for 30 min, and reacted for 24 h at 0–5 °C. The PT could be obtained after washing by deionized water under a centrifugation process. (2) The above fabricated 0.7 g PT was added into the 10 mL GO solution (10 mg mL^−1^) via an ultrasound process to receive a homogeneous solution. Whereafter, the PT/GO solution was injected into a polypropylene plastic pipe with a 3 mm diameter and subsequently treated at 110 °C for 12 h. After a hydrothermal reaction, the fabricated PTG gel fiber was taken out from the tube directly by a pouring process. (3) As a result, the PTG aerogel fiber could be formed by a freeze-drying method for 12 h, while the PTG fiber could be formed by vacuum drying at 60 °C for 12 h. The rGO, TG and PG fibers were fabricated via a similar method to the PTG fiber without PT, PANI particles and Ti_3_C_2_T_x_ flakes, respectively.

### 4.2. The Fabrication of an FSC

Firstly, we synthesized a 1 M H_2_SO_4_/PVA gel electrolyte by dissolving 10 g of PVA powder (Shanghai Macklin Biochemical Co, Ltd., Shanghai, China) in 100 mL of a 1 M H_2_SO_4_ (Shanghai Lingfeng Chemical Reagent Co., Ltd., 98 wt%, Shanghai, China) solution. Then, the two fibers (PTG aerogel fiber and rGO fiber) were used as the negative and positive electrodes and arranged in parallel. Finally, the gel electrolyte was coated over the electrodes to assemble a PTG aerogel fiber-based FSC and a rGO fiber-based FSC.

### 4.3. Material Characterization

The morphology and structure of the fibers were investigated by SEM (ZEISS, Sigma 300, Oberkochen, Germany) and TEM (FEI, Tecnai G2 F30, Hillsboro, OR, USA) with an EDS attachment for elemental analysis, while XRD (Bruker, D8 discover, Billerica, MA, USA) was used to examine the crystalline structure at a scanning rate of 2° min^−1^. The specific surface area and surface chemical states were further measured via a Micromeritics ASAP 2020 (Micromeritics, Norcross, GA, USA) and an Axis Ultra DLD spectrometer (Shimadzu, Kyoto City, Japan) for XPS, respectively.

### 4.4. Electrochemical Performance Measurements

We tested the electrochemical performance of the fibers on a CHI660 electrochemical workstation. For the three-electrode system, we used the fiber as the working electrode, an Ag/AgCl electrode as the reference, a carbon rod as the counter electrode, and a 1 M H_2_SO_4_ solution as the electrolyte. Cyclic voltammetry (CV) tests were carried out at a scan rate from 1 to 20 mV s^−1^ with potential windows of −0.2–0.4 V. Electrochemical impedance spectra (EISs) were measured at frequencies ranging from 0.01 to 100,000 Hz. Galvanostatic charge/discharge (GCD) measurements were surveyed at a current density from 1 to 10 A g^−1^. The symmetrical solid-state FSC was composed of PTG fibers and H_2_SO_4_/PVA gel electrolytes. To assemble the solid-sate FSC, PTG fibers, jointed with a copper wire via silver paint, were coated by H_2_SO_4_/PVA gel electrolytes prior to encapsulation into a heat-shrinkable tube in parallel.

## Data Availability

The data in this work are available from the corresponding author upon reasonable request.

## Supplementary Materials

The following supporting information can be downloaded at: https://www.mdpi.com/article/10.3390/gels12080690/s1, Figure S1. The surface SEM images of rGO fiber with low and high magnifications. Figure S2. The surface SEM images of TG fiber with low and high magnifications. Figure S3. The surface SEM images of PTG fiber with low and high magnifications. Figure S4. The cross-sectional SEM images of rGO fiber with low and high magnifications. Figure S5. The cross-sectional SEM images of TG fiber with low and high magnifications. Figure S6. The cross-sectional SEM images of PTG fiber with low and high magnifications. Figure S7. XPS survey spectrum of PTG fiber. Figure S8. The GCD curves of PTG aerogel fiber at the current density of 1–10 A g^−1^. Figure S9. The GCD curves of rGO fiber at the current density of 1–10 A g^−1^. Figure S10. The GCD curves of TG fiber at the current density of 1–10 A g^−1^. Figure S11. The GCD curves of PTG fiber at the current density of 1–10 A g^−1^. Figure S12. Capacitive and diffusion- controlled contributions to charge storage at a scan rate of 1 mV s^−1^. Figure S13. Capacitive and diffusion- controlled contributions to charge storage at a scan rate of 2 mV s^−1^. Figure S14. Capacitive and diffusion- controlled contributions to charge storage at a scan rate of 5 mV s^−1^. Figure S15. Capacitive and diffusion- controlled contributions to charge storage at a scan rate of 10 mV s^−1^. Figure S16. Capacitive and diffusion- controlled contributions to charge storage at a scan rate of 20 mV s^−1^. Figure S17. CV curves of rGO fiber based FSC at scan rate of 1–20 mV s^−1^. Figure S18. GCD curves of rGO fiber based FSC at current density of 1–3 A g^−1^. Table S1. The comparison of the capacitance of PTG electrode with previously reported literature. Table S2. The comparison of the capacitance of PTG aerogel fiber based FSC with previous reported literature. Table S3. The comparison of the energy density of PTG aerogel fiber based FSC with previously reported literature.
