# Supplementary material for: High-Performance Fiber-Shaped Supercapacitors Enabled by Polyaniline @ MXene Ti3C2Tx Hybrid Graphene Aerogel Fiber
Source: Gels. 2026 Aug 3;12(8):690. doi: 10.3390/gels12080690 (PMC13512521; doi:10.3390/gels12080690)
Supplement: Supplementary file 1 [file gels-12-00690-s001.zip › gels-4421935-supplementary.pdf]

Supporting information

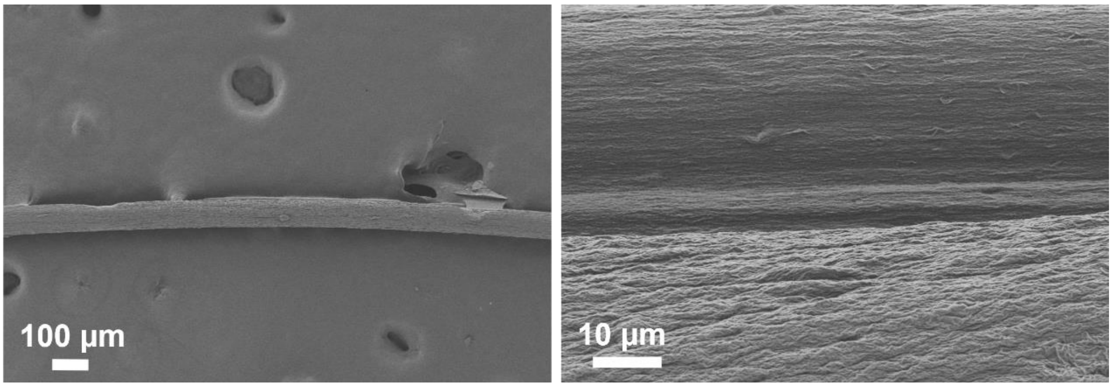

Figure S1. The surface SEM images of rGO fiber with low and high magnifications.

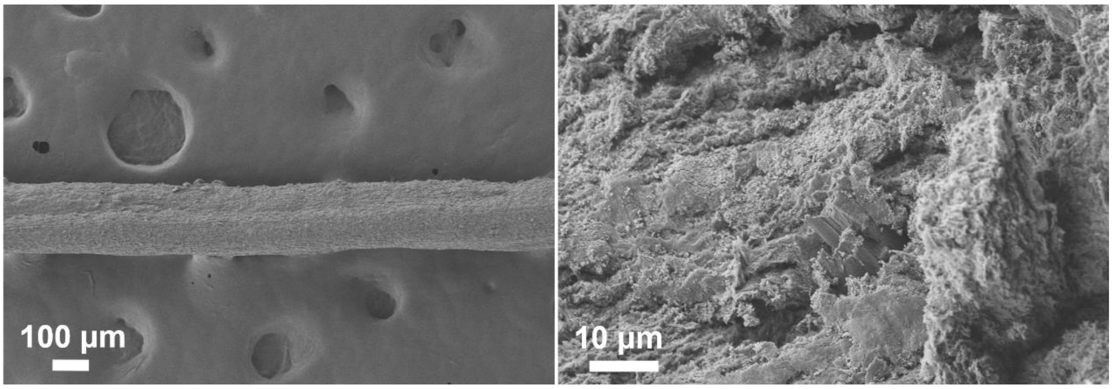

Figure S2. The surface SEM images of TG fiber with low and high magnifications.

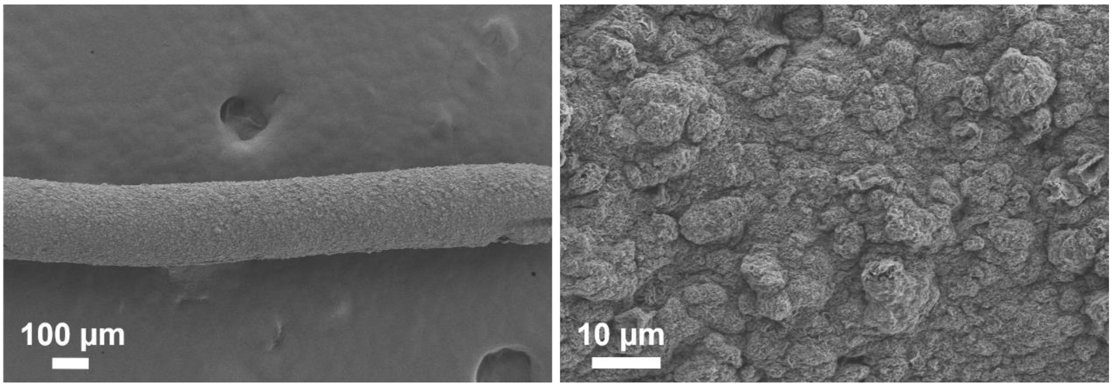

Figure S3. The surface SEM images of PTG fiber with low and high magnifications.

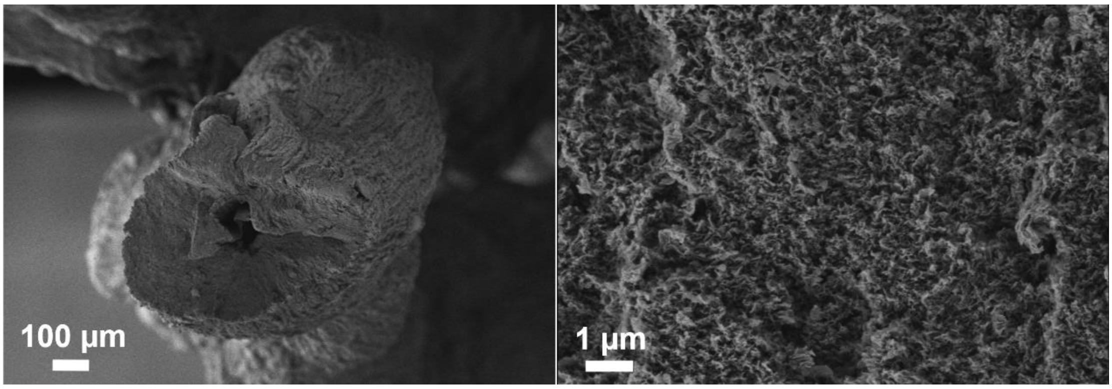

Figure S4. The cross-sectional SEM images of rGO fiber with low and high magnifications.

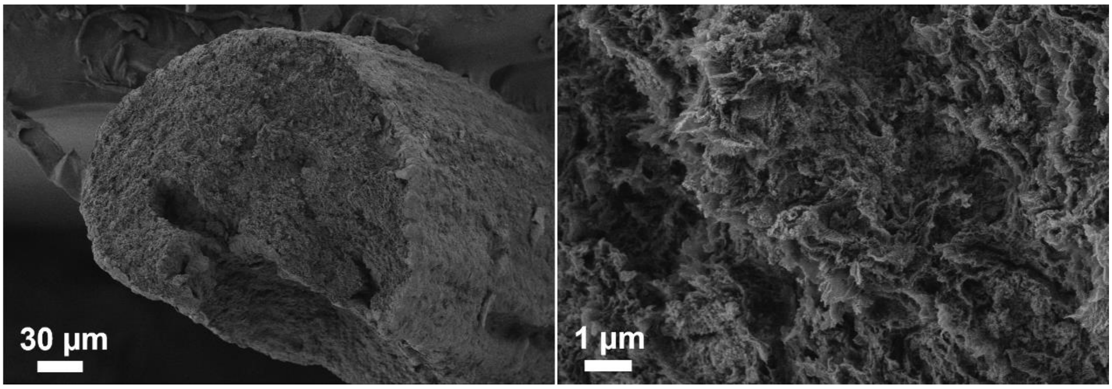

Figure S5. The cross-sectional SEM images of TG fiber with low and high magnifications.

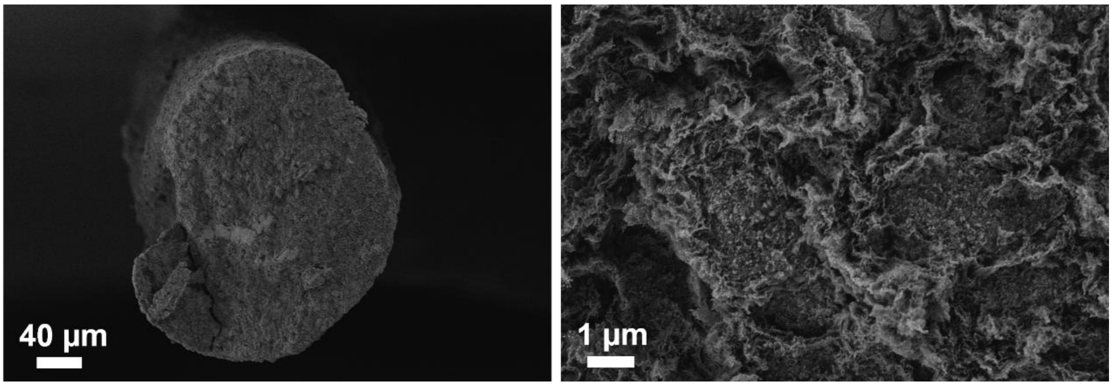

Figure S6. The cross-sectional SEM images of PTG fiber with low and high magnifications.

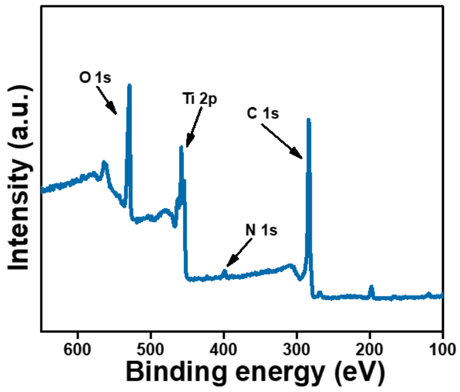

Figure S7. XPS survey spectrum of PTG fiber.

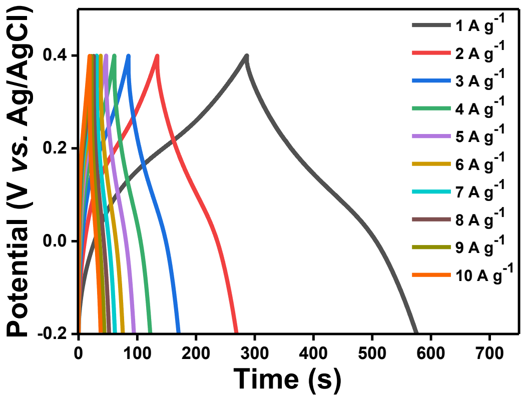

Figure S8. The GCD curves of PTG aerogel fiber at the current density of 1-10 A g^{-1}.

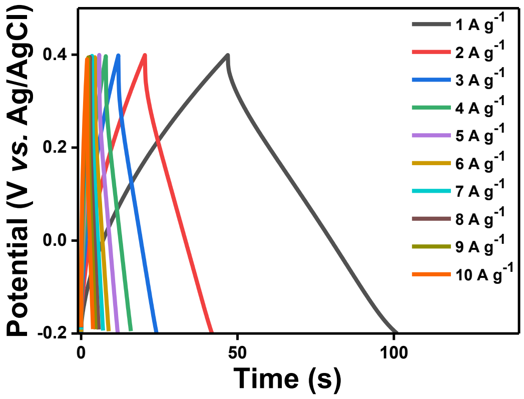

Figure S9. The GCD curves of rGO fiber at the current density of 1-10 A g^{-1}.

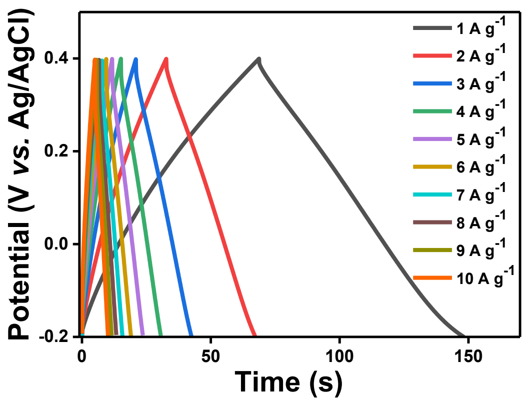

Figure S10. The GCD curves of TG fiber at the current density of 1-10 A g^{-1}.

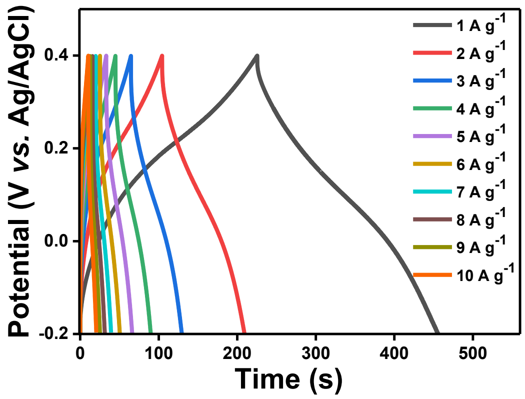

Figure S11. The GCD curves of PTG fiber at the current density of 1-10 A g^{-1}.

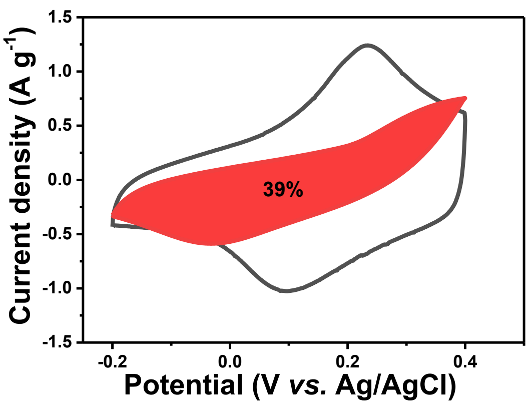

Figure S12. Capacitive and diffusion- controlled contributions to charge storage at a scan rate

of 1 mV s^{-1}.

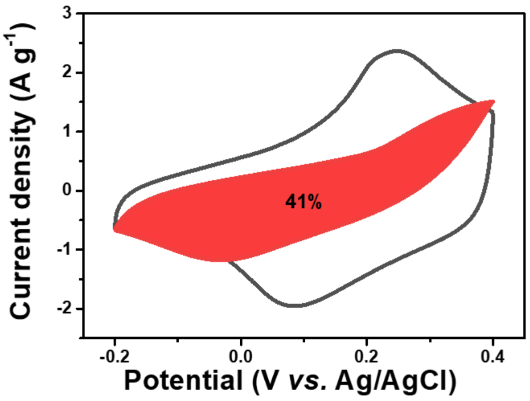

Figure S13. Capacitive and diffusion- controlled contributions to charge storage at a scan rate of 2 mV s^{-1}.

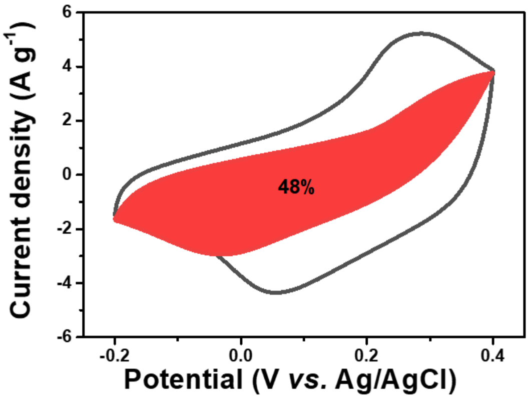

Figure S14. Capacitive and diffusion- controlled contributions to charge storage at a scan rate of 5 mV s^{-1}.

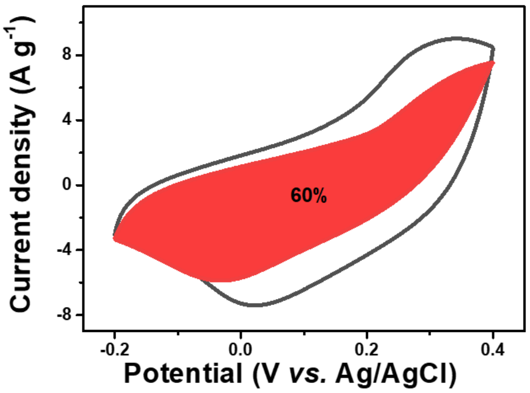

Figure S15. Capacitive and diffusion- controlled contributions to charge storage at a scan rate of 10 mV s^{-1}.

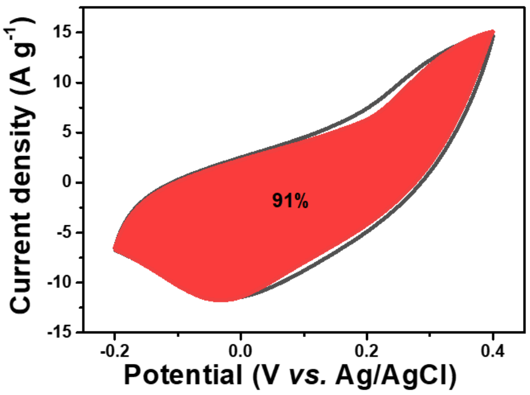

Figure S16. Capacitive and diffusion- controlled contributions to charge storage at a scan rate of 20 mV s^{-1}.

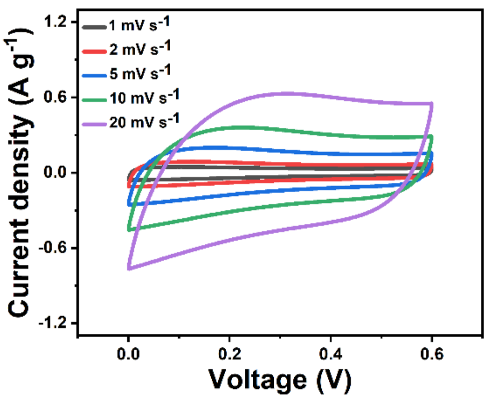

Figure S17. CV curves of rGO fiber based FSC at scan rate of 1-20 mV s^{-1}.

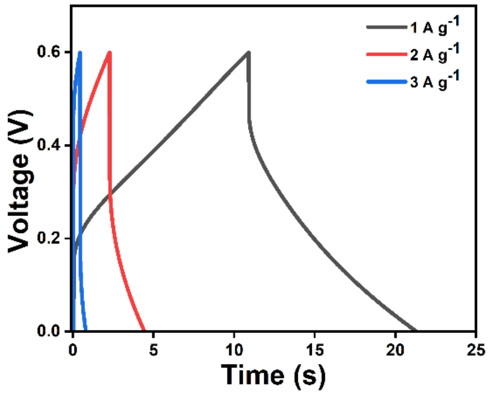

Figure S18. GCD curves of rGO fiber based FSC at current density of 1-3 A g^{-1}.

Table S1. The comparison of the capacitance of PTG electrode with previously reported literature.

Electrode	Electrolyte	Capacitance ($F\ g^{-1}$)	Cycling stability		Reference
			Cycles	Capacitance Retention (%)	
PTG aerogel fiber	1 M H_2SO_4	484.8 F g^{-1} (1 A g^{-1})	5000	85.2%	This work
PANI/MXene(V_2C)	1 M H_2SO_4	337.5 F g^{-1} (1 A g^{-1})	10000	97.6 %	[1]
CNT/PANI composite	1 M H_3PO_4	272.7 F g^{-1} (1 A g^{-1})	2000	90 %	[2]
MXene@ZIF-67	3 M KOH	97.10 F g^{-1} (1 A g^{-1})	N.R.	N.R.	[3]
rGO/MXene hybrid fiber	1 M H_2SO_4	195 F g^{-1} (0.1 A g^{-1})	7500	125 %	[4]
PANI/N-CNT@CNT fiber	1 M H_2SO_4	323.8 F g^{-1} (1 A g^{-1})	10000	92.1 %	[5]
Elastic fiber/CNTs/ PANI	PVA/H_3PO_4	255.5 F g^{-1} (1 A g^{-1})	10000	69 %	[6]
PANI@GO coated silk fabric	PVA/H_3PO_4	71.2 F g^{-1} (1 A g^{-1})	5000	87.4 %	[7]
Nitrogen-doped graphene/ polyacrylic acid/ polyaniline	H_2SO_4 -PVA	68 F g^{-1} (1 A g^{-1})	2000	83.2 %	[8]

composites					
CNT sheets coated on elastic fiber	PVA-H_3PO_4	41.4 F g^{-1} (0.1 A g^{-1})	1000	90 %	[9]
ZnO@CFT	1 M KOH	201 F g^{-1} (1 A g^{-1})	3000	90.32 %	[10]
C-Fe/PANI/Ni-GF	1 M $NaNO_3$	139.9 F g^{-1} (1 A g^{-1})	10000	91 %	[11]
MoS_2-rGO/ PEDOTNP/NF	3 M KOH	298 F g^{-1} (1 A g-1)	10000	93.17 %	[12]

Table S2. The comparison of the capacitance of PTG aerogel fiber based FSC with previous reported literature.

Electrode	Electrolyte	Capacitance (F g^{-1})	Cycling stability		Reference
			Cycles	Capacitance Retention (%)	
PTG aerogel fiber	PVA/H_2SO_4	275.6 F g^{-1} (1 A g^{-1})	5,000	86.6%	This work
CNTs/PANI/ Elastic fiber	PVA/H_3PO_4	255.5 F g^{-1} (1 A g^{-1})	10,000	69 %	[6]
Hydrothermally activated graphene fiber	PVA/H_2SO_4	244 F g^{-1} (0.1 A g^{-1})	300	92.2 %	[13]
MCNF- mesoporous carbon nanofibers	PVA/H_2SO_4	216.6 F g^{-1} (1 A g^{-1})	3000	96.7 %	[14]
PPy/MWCNT/cotton fabric	PVA/H_3PO_4	201.99 F g^{-1} (1.8 A g^{-1})	375	93 %	[15]
PANI@GO coated silk fabric	PVA/H_3PO_4	71.2 F g^{-1} (1 A g^{-1})	5000	87.4 %	[7]
CNT sheets coated on elastic fiber	PVA/H_3PO_4	41.4 F g^{-1} (0.1 A g^{-1})	1000	90 %	[9]

Table S3. The comparison of the energy density of PTG aerogel fiber based FSC with previously reported literature.

Electrode	Power density (W kg^{-1})	Energy density (Wh kg^{-1})	Reference
PTG aerogel fiber	202.4	3.44	**This work**
Carbon fiber/Polyester	12.57	0.106	[16]

Polypyrrole-MXene	41.1	1.3	[17]
CNT-$NiCo_2O_4$	18.5	3.2	[18]
MCNT/bamboo joint	157	2.27	[19]
PANI/MXene@PET	3.8	0.69	[20]
CCY-MnO_2	47.7	0.17	[21]

Reference

[1] X. Wang, D. Zhang, H. Zhang, L. Gong, Y. Yang, W. Zhao, S. Yu, Y. Yin, D. Sun, In situ polymerized polyaniline/MXene (V_2C) as building blocks of supercapacitor and ammonia sensor self-powered by electromagnetic-triboelectric hybrid generator, Nano Energy 88 (2021) 106242.

[2] S. Pan, H. Lin, J. Deng, P. Chen, X. Chen, Z. Yang, H. Peng, Novel Wearable Energy Devices Based on Aligned Carbon Nanotube Fiber Textiles, Adv. Energy Mater. 5 (2014) 1401438.

[3] C. Liu, W. Feng, Y. Bai, H. Pang, Compositing MXenes with hierarchical ZIF-67/cobalt hydroxideviacontrollablein situetching for a high-performance supercapacitor, Inorg. Chem. Front. 9 (2022) 5463-5468.

[4] Z. Wang, Y. Chen, M. Yao, J. Dong, Q. Zhang, L. Zhang, X. Zhao, Facile fabrication of flexible rGO/MXene hybrid fiber-like electrode with high volumetric capacitance, J. Power Sources 448 (2020) 227398.

[5] J. Tian, N. Cui, P. Chen, K. Guo, X. Chen, High-performance wearable supercapacitors based on PANI/N-CNT@CNT fiber with a designed hierarchical core-sheath structure, J. Mater. Chem. A 9 (2021) 20635-20644.

[6] X. Chen, H. Lin, J. Deng, Y. Zhang, X. Sun, P. Chen, X. Fang, Z. Zhang, G. Guan, H. Peng, Electrochromic Fiber-Shaped Supercapacitors, Adv. Mater. 26 (2014) 8126-8132.

[7] M. Khairy, R. Kamal, M.A. Mousa, Preparation and physical properties of conductive silk fabrics used in wearable clothes and flexible supercapacitors, J. Ind. Text. 52 (2022) 1–21.

[8] Y. Wang, S. Tang, S. Vongehr, J. Ali Syed, X. Wang, X. Meng, High-Performance Flexible Solid-State Carbon Cloth Supercapacitors Based on Highly Processible N-Graphene Doped Polyacrylic Acid/Polyaniline Composites, Sci. Rep. 6 (2016) 12883.

[9] Z. Yang, J. Deng, X. Chen, J. Ren, H. Peng, A Highly Stretchable, Fiber-Shaped Supercapacitor, Angew. Chem. Int. Edit. 52 (2013) 13453-13457.

[10] Q. Abbas, M. Javed, A. Ahmad, S. Siyal, I. Asim, R. Luque, M. Albaqami, A. Tighezza, ZnO Nano-Flowers Assembled on Carbon Fiber Textile for High-Performance Supercapacitor’s Electrode, Coatings 11 (2021) 1337.

[11] M.N. Rantho, M.J. Madito, N. Manyala, High-performance symmetric supercapacitor device based on carbonized iron-polyaniline/nickel graphene foam, J. Alloy. Compd. 819 (2020) 152993.

[12] D. Sarmah, A. Kumar, Symmetric Supercapacitors with layer-by-layer Molybdenum disulfide - reduced graphene oxide structures and poly(3,4-ethylenedioxythiophene)

nanoparticles nanohybrid electrode, J. Energy Storage 35 (2021) 102289.
[13] Z. Li, T. Huang, W. Gao, Z. Xu, D. Chang, C. Zhang, C. Gao, Hydrothermally Activated Graphene Fiber Fabrics for Textile Electrodes of Supercapacitors, ACS Nano 11 (2017) 11056-11065.
[14] Y. Li, W. Ou-Yang, X. Xu, M. Wang, S. Hou, T. Lu, Y. Yao, L. Pan, Micro-/mesoporous carbon nanofibers embedded with ordered carbon for flexible supercapacitors, Electrochim. Acta 271 (2018) 591-598.
[15] S. Huang, P. Chen, W. Lin, S. Lyu, G. Chen, X. Yin, W. Chen, Electrodeposition of polypyrrole on carbon nanotube-coated cotton fabrics for all-solid flexible supercapacitor electrodes, RSC Adv. 6 (2016) 13359-13364.
[16] B.K. Deka, A. Hazarika, J. Kim, Y.-B. Park, H.W. Park, Multifunctional CuO nanowire embodied structural supercapacitor based on woven carbon fiber/ionic liquid–polyester resin, Compos. Part A 87 (2016) 256-262.
[17] J. Yan, Y. Ma, C. Zhang, X. Li, W. Liu, X. Yao, S. Yao, S. Luo, Polypyrrole–MXene coated textile-based flexible energy storage device, RSC Adv. 8 (2018) 39742-39748.
[18] Y. Hu, Q. Wang, S. Chen, Z. Xu, M. Miao, D. Zhang, Flexible Supercapacitors Fabricated by Growing Porous $NiCo_2O_4$ In Situ on a Carbon Nanotube Film Using a Hyperbranched Polymer Template, ACS Appl. Energy Mater. 3 (2020) 4043-4050.
[19] Y. Xu, Y. Zhang, D. Zhang, J. Ma, W. Yi, J. Zhang, H. Shi, Synthesis of multiwall carbon nanotubes via an inert atmosphere absent autogenetic-pressure method for supercapacitor, J. Energy Storage 26 (2019) 100995.
[20] X. Zheng, Y. Wang, W. Nie, Z. Wang, Q. Hu, C. Li, P. Wang, W. Wang, Elastic polyaniline nanoarrays/MXene textiles for all-solid-state supercapacitors and anisotropic strain sensors, Compos. Part A 158 (2022) 106985.
[21] S. Ullah, J. Yu, H. Liu, W. Iqbal, B. Yang, C. Li, C. Zhu, J. Xu, Fabrication of MnO_2-carbonized cotton yarn derived hierarchical porous active carbon flexible supercapacitor electrodes for potential applications in cable-type devices, Appl. Surf. Sci. 487 (2019) 180-188.
